# Native Human Monoclonal Antibodies with Potent Cross-Lineage Neutralization of Influenza B Viruses

**DOI:** 10.1128/AAC.02269-17

**Published:** 2018-04-26

**Authors:** Adam Vigil, Angeles Estélles, Lawrence M. Kauvar, Scott K. Johnson, Ralph A. Tripp, Michael Wittekind

**Affiliations:** a ContraFect Corporation, Yonkers, New York, USA; b Trellis Bioscience, Menlo Park, California, USA; c University of Georgia, Department of Infectious Disease, Athens, Georgia, USA

**Keywords:** IBV, influenza, monoclonal antibodies

## Abstract

Although antibodies that effectively neutralize a broad set of influenza viruses exist in the human antibody repertoire, they are rare. We used a single-cell screening technology to identify rare monoclonal antibodies (MAbs) that recognized a broad set of influenza B viruses (IBV). The screen yielded 23 MAbs with diverse germ line origins that recognized hemagglutinins (HAs) derived from influenza strains of both the Yamagata and Victoria lineages of IBV. Of the 23 MAbs, 3 exhibited low expression in a transient-transfection system, 4 were neutralizers that bound to the HA head region, 11 were stalk-binding nonneutralizers, and 5 were stalk-binding neutralizers, with 4 of these 5 having unique antibody sequences. Of these four unique stalk-binding neutralizing MAbs, all were broadly reactive and neutralizing against a panel of multiple strains spanning both IBV lineages as well as highly effective in treating lethal IBV infections in mice at both 24 and 72 h postinfection. The MAbs in this group were thermostable and bound different epitopes in the highly conserved HA stalk region. These characteristics suggest that these MAbs are suitable for consideration as candidates for clinical studies to address their effectiveness in the treatment of IBV-infected patients.

## INTRODUCTION

Influenza B viruses (IBV) have two antigenically differentiated lineages, termed Yamagata and Victoria (1, 2), which differ enough to warrant the inclusion of two IBV-specific components in quadrivalent influenza vaccines (3). Vaccination against IBV is only a partially effective means of protection, as IBV is subject to antigenic drift among circulating strains prevalent in successive seasons, necessitating annual vaccine reformulation. Influenza vaccine effectiveness can be further compromised when trivalent vaccines, which have only a single IBV-specific component and fail to cover both IBV lineages, are used (4–6). While IBV does not cause pandemics, as there are no known naturally occurring nonhuman IBV reservoirs permitting extensive antigenic shift, in some years IBV can be the predominant circulating influenza strain in certain geographic regions (4, 7, 8) and IBV infections can be as severe as or even more severe than infections due to influenza A viruses (IAV) (9). IBV can cause serious life-threatening infections in pediatric populations. For example, in the 2010-2011 season, IBV caused 25% of the influenza cases, while it was the cause of 38% of the pediatric deaths (10). The marketed anti-influenza neuraminidase inhibitor oseltamivir is less effective in treating IBV infections than IAV infections (11), highlighting the imperative for the development of new effective anti-IBV treatments for this prevalent and sometimes deadly infection.

Several attempts to define strain-independent epitopes on the hemagglutinin (HA) surface glycoprotein of influenza viruses have been made for vaccine candidates or therapeutic monoclonal antibodies (MAbs) (reviewed in reference 12). Neutralization of viruses from both group 1 and group 2 strains of IAV by single cross-reactive MAbs has been reported (13, 14), although these generally have a lower overall potency than group-specific MAbs (15–17). These investigative efforts have focused attention on the stalk region of the HA protein, which is essential for virus-cell fusion and is more conserved than the HA globular head domain. For the fusion site in the stalk to be functional, the HA_0_ primary protein must first be activated by proteolytic cleavage into the HA_1_ and HA_2_ subunits, which remain coupled by disulfide bonds, thereby facilitating the conformational change of HA necessary for fusion of the viral and cellular membranes (18). The ability of MAbs to block the conformational change of HA at the low pH of the endosomal compartment appears to be the key attribute of stalk-binding neutralizing MAbs responsible for preventing influenza viral infectivity (19).

Many of the MAbs to the HA stalk region have been derived from *in vitro* phage display antibody libraries (16, 17, 20). Alternatively, in the case of IAV, high-quality MAbs were obtained directly from memory B cells derived from people vaccinated against or naturally infected with influenza virus (13, 15, 21). This natural repertoire of affinity-matured MAbs has provided effective immunity against influenza in model systems, making them attractive as a source for therapeutic candidates. Human MAbs in general have had low failure rates in phase 1 clinical trials (22), and native human MAbs may have an even lower risk, having been safely produced in at least the one human from whom it was cloned. Memory B cells are of particular interest for their potential to be an enriched source of B cells that display reactivity against the multiple strains of influenza virus encountered over decades. However, blood samples from human donors can vary widely in their frequency of high-quality neutralizing antibodies for a particular pathogen, and the frequency can be low (15).

The CellSpot technology provides the ability to screen for rare human antibodies and has been used to generate clinical candidate MAbs against respiratory syncytial virus (23), cytomegalovirus (24), a bacterial target implicated in antibiotic resistance (25), and immune-regulating MAbs (26). This approach uses antigen-independent stimulation of B cells to induce the secretion of IgG, which is captured as a microscopic footprint around the cell (∼150 μm in diameter). Millions of these footprints (i.e., cell spots), each comprising a single MAb, are then probed in parallel with multiple antigens on distinguishable fluorescent beads. A computerized microscope tabulates the antigen specificity for the MAb in each B cell footprint by counting the number of each type of captured bead (up to ∼10,000 beads can bind to one cell spot, providing a dynamic range of ∼1.5 logs for each analyte). Following isolation of the rare B cells with favorable specificity profiles, heavy and light chains are cloned by single-cell reverse transcription-PCR (RT-PCR), and MAbs are expressed by transient transfection in HEK293 cells. The complete assay is conducted on a time scale compatible with the limited *in vitro* lifetime of human B cells, thereby enabling isolation of the mRNAs encoding the MAb heavy and light chain variable regions from rare favorable cells.

Applying the CellSpot technology to HA from widely divergent IAV subtypes yielded strain-independent MAbs to group 1 and to group 2 IAVs (15). When expressed as an intact recombinant IgG1, these native human anti-IAV MAbs reliably recapitulated the binding properties observed in the primary assay. We now report the application of this technology to isolate MAbs that bind and neutralize IBV with the aim of discovering new antiviral agents.

## RESULTS

Our aim was to discover anti-IBV MAbs that have the following qualities: (i) broadly reactive against circulating IBV strains by targeting the conserved HA stalk region, (ii) high-affinity binding *in vitro*, (iii) high potency *in vivo* (as assessed in a murine IBV infection model), (iv) suitable for human use, and (v) structurally stable.

### Primary screen.

To achieve these goals, we applied the CellSpot technology to anonymized human blood samples. To identify broadly reactive anti-IBV MAbs, we used HA antigens from representative members of the two major lineages of IBV strains: B/Florida/04/2006 (Yamagata lineage) and B/Malaysia/2506/2004 (Victoria lineage) (27). Eight blood donors were surveyed, with a total of approximately 2.5 million memory B cells being screened both in dedicated IBV screens and as controls in screens for other antigens. Memory B cell (CD19^+^/CD27^+^) abundance was measured and adjusted to equal levels in each screen. In the primary screen, for most blood samples the average frequency at which memory B cells secreted a MAb against IBV HA was 2 in 10,000, which was about 10-fold higher than what we previously found when screening for MAbs against IAV HAs. Of these anti-IBV MAbs, about half showed cross-reactivity to HAs from both the B/Florida/04/2006 and B/Malaysia/2506/2004 strains. For further characterization, 23 MAbs were expressed in HEK293 cells and confirmed as being double positive for both IBV HAs.

### Secondary screens.

Of the 23 initial antibody clones examined, 20 were produced at sufficiently high levels (at least 20 mg/liter) in a mammalian HEK293 cell transient expression system to warrant further investigation. The germ line origins for the sequences of these 20 MAbs are diverse, indicating that the CellSpot process surveys a wide sampling of the natural human repertoire (Table 1). All of the 20 MAbs discovered in this study were derived from germ line sequences different from those from which the previously characterized IBV-specific MAbs, 5A7 (28) and CR8033, CR8071, and CR9114 (14), were derived. These 20 antibodies were further tested for their ability to neutralize two IBV strains (B/Victoria/2/1987 and B/Yamagata/16/1988) that were different from the strains from which the HAs used in the CellSpot screen were derived. Eleven MAbs failed to neutralize one or both of these two IBV strains and were dropped from further consideration.

**TABLE 1:** Characterization of native human anti-HA IBV MAbs

MAb classification	MAb	Expression in HEK293 cells^*a*^	HI activity^*b*^		Neutralization in MDCK cells^*c*^		Predicted binding site, mode	Germ line^*d*^	
			IBV Victoria	IBV Yamagata	IBV Victoria	IBV Yamagata		HC	LC
Low expression	TRL798	−	ND^*f*^	ND	ND	ND	ND	IGHV1-2*02	IGKV1-6*01
	TRL834	−	ND	ND	ND	ND	ND	IGHV1-69*12	IGLV1-44*01
	TRL851	−	ND	ND	ND	ND	ND	IGHV5-51*01	IGKV2D-28*01
									
HA head neutralizing	TRL809	+	+/−	+	+	+	Head, neut^*g*^	IGHV3-30-3*02	IGKV4-1*01
	TRL823	+	+	+	+	+	Head, neut	IGHV3-43*02	IGKV1-39*01
	TRL832	+	+	+	+	+	Head, neut	IGHV4-59*02	IGLV3-1*01
	TRL833	+	+	+	+	+	Head, neut	IGHV4-31*03	IGLV3-1*01
									
HA stalk nonneutralizing	TRL784	+	−	−	−	−	Stalk, no neut	IGHV1-69*12	IGLV3-21*02
	TRL799	+	−	−	−	−	Stalk, no neut	IGHV1-2*02	IGKV2D-28*01
	TRL811	+	−	−	−	−	Stalk, no neut	IGHV3-30*13	IGKV1-17*01
	TRL812	+	+/−	−	+/−	+/−	Stalk, no neut	IGHV3-33*01	IGKV3-15*01
	TRL813	+	−	−	+/−	+/−	Stalk, no neut	IGHV3-73*01	IGKV1-5*03
	TRL835	+	−	−	−	−	Stalk, no neut	IGHV5-51*01	IGKV2D-28*01
	TRL837	+	−	+/−	−	+	Stalk, no neut	IGHV1-69*12	IGKV3-20*01
	TRL841	+	−	−	−	−	Stalk, no neut	IGHV3-30*18	IGKV3-15*01
	TRL842	+	−	−	−	−	Stalk, no neut	IGHV3-30*18	IGLV3-21*02
	TRL846	+	−	−	−	−	Stalk, no neut	IGHV3-23*04	IGKV3-NL1*01
	TRL856	+	ND	−	−	−	Stalk, no neut	IGHV3-74*01	IGLV3-21*02
									
HA stalk neutralizing	TRL845	+	−	−	+	+	Stalk, neut	IGHV3-48*02	IGKV1-9*01
	TRL847	+	−	−	+	+	Stalk, neut	IGHV3-48*02	IGKV1-9*01
	TRL848	+	−	−	+	+	Stalk, neut	IGHV3-30*18	IGLV3-21*02
	TRL849	+	−	−	+	+	Stalk, neut	IGHV4-31*03	IGKV3-15*01
	TRL854	+	−	−	+	+	Stalk, neut	IGHV1-2*02	IGKV2D-28*01
									
Previously published MAbs^*e*^	5A7	+	ND	ND	ND	ND	Stalk, neut	IGHV3-33*01	IGLV1-47*02
	CR8033	ND	ND	ND	ND	ND	Head, neut	IGHV3-9*01	IGKV3-20*01
	CR8071	ND	ND	ND	ND	ND	Head, neut	IGHV1-18*01	IGLV1-47*01
	CR9114	+	ND	ND	ND	ND	Stalk, no neut	IGHV1-69*06	IGLV1-44*01

a Expression in HEK293 cells was classified as an expression level of >20 mg/liter (+) and an expression level of <20 mg/liter (−).

b Hemagglutination inhibition activities against strains B/Victoria/2/1987 and B/Yamagata/16/1988 at effective MAb concentrations of 1.5 μg/ml (+), 200 μg/ml (+/−), and >200 μg/ml (−).

c *In vitro* neut, *in vitro* neutralization IC_50_s against strains B/Victoria/2/1987 and B/Yamagata/16/1988 at MAb concentrations of ≤10 μg/ml (+) and >10 μg/ml (−).

d Germ line families for the heavy chain (HC) and light chain (LC) are listed using the IMGT nomenclature (www.imgt.org).

e MAb 5A7 is described in reference 28, and the CR MAbs are described in reference 14.

f ND, not determined.

g neut, neutralization.

For IAVs, it is generally more difficult to generate escape mutants against MAbs that bind the conserved stalk region of HA than to generate escape mutants against MAbs that bind the head region (29). Therefore, we sought to identify panreactive anti-IBV MAbs that bound to the conserved stalk region. Of the nine remaining broadly reactive MAbs, four were found to exhibit inhibitory activity in a hemagglutination inhibition (HI) assay (using the same strains used for the neutralization screen). A positive reading in the HI assay depends on the interaction of the globular head of HA with sialic acid receptors on the surface of red blood cells (30). Therefore, these four HI-positive MAbs were considered presumptive HA head-binding MAbs and were removed from further consideration.

Of the remaining final five lead candidate MAbs (TRL845, TRL847, TRL848, TRL849, TRL854), most have unique heavy and light chain sequences and likely arose from independent B cell lineages. The exceptions were MAbs TRL845 and TRL847 (derived from the same donor), which were closely related at the sequence level (Table 2). Identification of two related clones in the final five lead candidates was indicative of the reproducibility of the CellSpot assay and the subsequent screening process.

**TABLE 2:** Lead MAb variable region sequences^*a*^

MAb	Heavy chain				Light chain			
	Heavy chain gene	HCDR1 sequence	HCDR2 sequence	HCDR3 sequence	Light chain gene	LCDR1 sequence	LCDR2 sequence	LCDR3 sequence
TRL845	IGHV3-48*02	GFTFCRYI	ISDTSRTI	ARDPDTPFVRAFDS	IGKV1-9*01	QLISSY	AAS	PPLNSYPPIT
TRL847	IGHV3-48*02	GFTFSRFS	ISDTGRTI	ARDPDTPFVRAFDS	IGKV1-9*01	QVISSY	AAS	QQLTTYPPIT
TRL848	IGHV3-30*18	GFSLWTSG	MSYDETKK	AKPRLDYLDYFHAADS	IGLV3-21*02	YIGSKS	DDS	CQVWETSEDLWV
TRL849	IGHV4-31*03	GGSISNGGYH	IYYRGST	ARMPLANYDLLTGLYIGAFDL	IGKV3-15*01	QSVNRN	DAS	QQYDKWPPG
TRL854	IGHV1-2*02	GYTFTAYH	INPNSGAT	ATDIVVERDASLGGFNSYGMDV	IGKV2D-28*01	QSLLHSNGYNH	LAS	MQSLQTSIT

a The amino acid sequences (in single-letter code) of the three complementarity-determining regions of the MAb heavy and light chains (HCDR1 to HCDR3 and LCDR1 to LCDR3, respectively) are listed. The IMGT designations of the germ line antibody sequences are listed for the heavy and light chains for each MAb.

### Binding constant determination.

**FIG 1:**
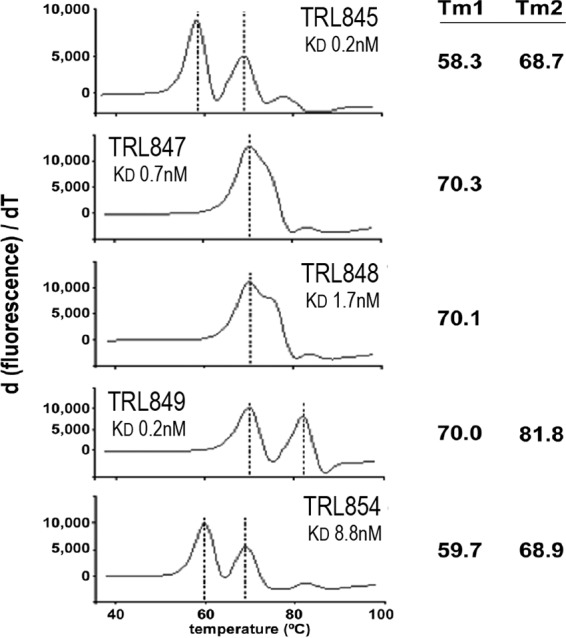
Thermostabilities and affinities of lead MAbs. The melting temperatures (*T_m_*; in degrees Celsius) of the MAbs were determined by binding of SYPRO Orange fluorescent dye (Thermo Fisher). The first derivative of the melting curve is plotted to highlight the *T_m_*. The observed biphasic *T_m_* for the MAbs are due to the separate contributions of the heavy and light chains. Dissociation constants (*K_D_*) were measured with a ForteBio Octet biosensor. T, time.

The affinities of these five MAbs for HA of B/Florida/04/2006 were measured using the ForteBio Octet biosensor assay (Fig. 1), with TRL845 and TRL849 being the highest-affinity candidates, which had equilibrium dissociation constant (*K_D_*) values of ≤200 pM. The broad reactivities of the five MAbs against different IBV strains were illustrated by binding to HAs from three lineage Yamagata strains and three lineage Victoria strains (Table 3). The HA of IBV is sufficiently different from that of IAV that the previously described high-affinity MAbs TRL053 and TRL579 (15), directed against the HAs of the H1N1 and H3N2 IAV subtypes, respectively, show negligible cross-reactivity to IBV and vice versa.

**TABLE 3:** Binding of anti-HA stalk-neutralizing MAbs to influenza A and B virus HAs

Influenza A and B virus HA	ELISA signal^*a*^						
	Anti-IBV					Anti-IAV	
	TRL845	TRL847	TRL848	TRL849	TRL854	TRL053	TRL579
IBV Victoria lineage HA							
B/Brisbane/60/2008	+++	+++	+++	+++	+++	−	−
B/Malaysia/2506/2004	+++	+++	+++	+++	+++	−	−
B/Victoria/2/1987	+++	+++	+++	+++	+++	−	−
IBV Yamagata lineage HA							
B/Florida/4/2006	+++	+++	+++	+++	+++	−	−
B/Massachusetts/02/2012	+++	+++	+++	+++	+++	−	−
B/Wisconsin/1/2010	+++	+++	+++	+++	+++	−	−
							
IAV (H1N1) group 1 HA							
A/California/07/2009	−	−	−	−	−	+++	−
							
IAV (H3N2) group 2 HA							
A/Sydney/05/1997	−	−	−	−	−	−	+++

a The ELISA signals are represented as negative (−; i.e., the background level) or strongly positive (+++; i.e., corresponding to 18 to 21 times the background level). Anti-IAV control MAbs are previously described antibodies TRL053 and TRL579 (15).

### Thermal stability measurements.

The thermal stabilities of the five MAbs were assessed by differential scanning fluorimetry (31). The MAbs were slowly heated from 25°C to 99°C in the presence of the fluorescent dye SYPRO Orange, for which the fluorescence increases as the dye binds to hydrophobic residues exposed when the protein unfolds. As shown in Fig. 1, the melting temperature (*T_m_*) for all of the MAbs was 57°C or above. Interestingly, even though TRL845 and TRL847 are derived from the same germ line sequences and share a high degree of overall sequence identity, TRL847 is significantly more thermostable. The biphasic melting curve seen for most of the MAbs is a common phenomenon, with heavy and light chains differing in their stability (32).

### Epitope mapping.

**FIG 2:**
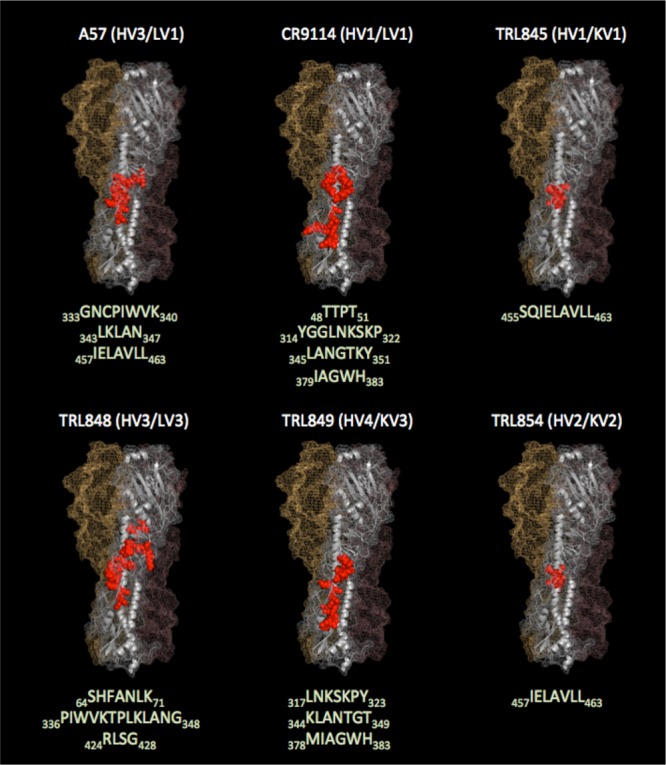
Epitopes determined by CLIPS analysis mapped onto the IBV trimeric HA structure. IBV-specific MAbs (2 from the literature and 4 from this study, with the germ line families being noted) all bind to the HA stalk region, with the specific peptide sequences recognized by each MAb being listed (the numbering scheme is based on the HA from B/Lee/40/1940). Those amino acids are shown in red using a space-filling representation for the surface, with a ribbon representation being used for the backbone. For clarity, the MAb-binding residues on only one of the three monomers that make up the HA homotrimer are shown. Graphic images were made using the PyMOL molecular graphics system (Schrödinger LLC).

The epitopes for four independent MAbs (excluding TRL847) were determined using the chemical linkage of peptides onto scaffolds (CLIPS) technology (33), comprising >6,500 linear and constrained synthetic peptide fragments (5 to 30 residues long) derived from the HA stalk sequence of B/Lee/40/1940. In addition, two published IBV stalk-binding MAbs, 5A7 (28) and CR9114 (14), were similarly analyzed. Figure 2 shows surface and backbone trace representations of the trimeric IBV HA structure, with the binding peptide segments indicated in red. Despite the fact that the heavy and light chains of all the MAbs analyzed in the CLIPS study whose results are shown in Fig. 2 were derived from completely different germ line sequences, convergent patterns of peptide recognition were observed in some cases. For example, TRL845 and TRL854 displayed nearly indistinguishable binding patterns. Also, TRL849 and CR9114 recognized a related set of peptides, even though they are very divergent at the sequence level and they exhibit widely different *in vivo* activities.

**FIG 3:**
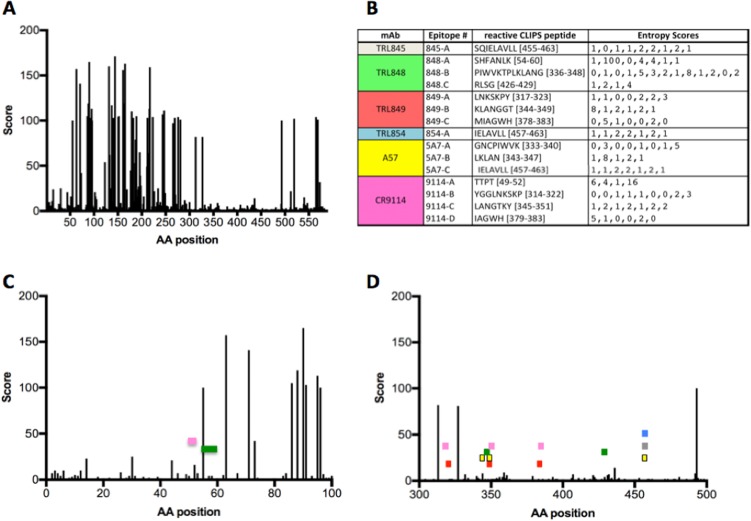
MAb epitopes map to HA segments with low sequence position diversity scores. (A, C, and D) The entropy scores (Score) at each HA residue position for the full-length HA (A) and expanded views of HA segments from amino acids (AA) 1 to 100 (C) and 300 to 500 (D). The segments of HA residues corresponding to the MAb epitopes (listed in panel B and Fig. 2) are illustrated by colored rectangles in panels C and D, following the coloring scheme indicated in panel B. (B) CLIPS peptide sequences that define each epitope and the corresponding entropy scores at each residue position.

To investigate the extent of HA sequence conservation for each MAb epitope, the entropy scores at each position were determined and mapped against an alignment of all currently available full-length IBV HA sequences isolated from human subjects (2,543 HA sequences) (Fig. 3). The epitopes of the four unique neutralizing stalk-binding MAbs obtained in this study, as well as two anti-IBV HA MAbs from the literature (14, 28), all mapped to HA sequences with low entropy scores within the HA alignment, indicating that all the MAbs recognize epitopes that are comprised of highly conserved elements within the HA structure. This high degree of sequence conservation at the HA epitope sites responsible for binding to the MAbs suggests that the MAbs are likely to bind and neutralize the vast majority of IBV strains circulating in the human population.

The only epitope corresponding to an HA segment encompassing a residue position with an entropy score above 16 (on a scale of from 1 to 232) is the first epitope of MAb TRL848 (HA residues S_54_H_55_F_56_A_57_N_58_L_59_K_60_). In this case, the high-entropy position is HA sequence position 55 (score = 100), where there is either a histidine or a phenylalanine for Victoria and Yamagata strains, respectively. Since TRL848 binds tightly to strains from both lineages (Table 3), residues other than position 55 within this epitope must be largely responsible for binding the TRL848 MAb. The other epitope segments display very low entropy scores at each of the corresponding HA residue positions (Fig. 3B).

### *In vitro* neutralization.

The IBV-neutralizing activities of MAbs TRL845, TRL848, and TRL849 were titrated to determine the 50% inhibitory concentrations (IC_50_s) in a plaque formation assay performed on MDCK cells (34) infected with either the B/Florida/04/2006 (Yamagata lineage) or B/Malaysia/2506/2004 (Victoria lineage) strain (Table 4). MAb TRL849 was additionally tested against three more strains and found to neutralize the Victoria lineage B/Nevada/3/2011 strain as well as two older IBV strains (B/Great Lakes/1954, B/Taiwan/2/1962), whose isolations predate the Yamagata/Victoria lineage divergence (1, 2).

**TABLE 4:** Neutralization IC_50_s for MAbs against IBV strains

IBV lineage	IBV strain	IC_50_ (mg/ml [95% CI])^*a*^		
		TRL845	TRL848	TRL849
Yamagata	B/Florida/04/2006	8.2 (6.1–11.3)	4.7 (2.9–8.0)	9.7 (6.7–14.6)
Victoria	B/Malaysia/2506/2004	1.2 (0.2–6.6)	1.1 (0.2–5.1)	1.4 (0.4–5.1)
Victoria	B/Nevada/03/2011			1.7 (0.3–9.4)
Pre-Yamagata/Victoria lineage split	B/Taiwan/2/1962			1.5 (0.3–8.9)
Pre-Yamagata/Victoria lineage split	B/Great Lakes/1954			25.7 (7.0^*b*^)

a The 50% inhibitory concentration (IC_50_) values were determined from data obtained from the plaque formation assay performed on MDCK cells (34). CI, confidence interval.

b The upper limit of the 95% confidence interval for the TRL849 IC_50_ against B/Great Lakes/1954 is undefined due to insufficient data for concentrations above the IC_50_.

### *In vivo* activity.

**FIG 4:**
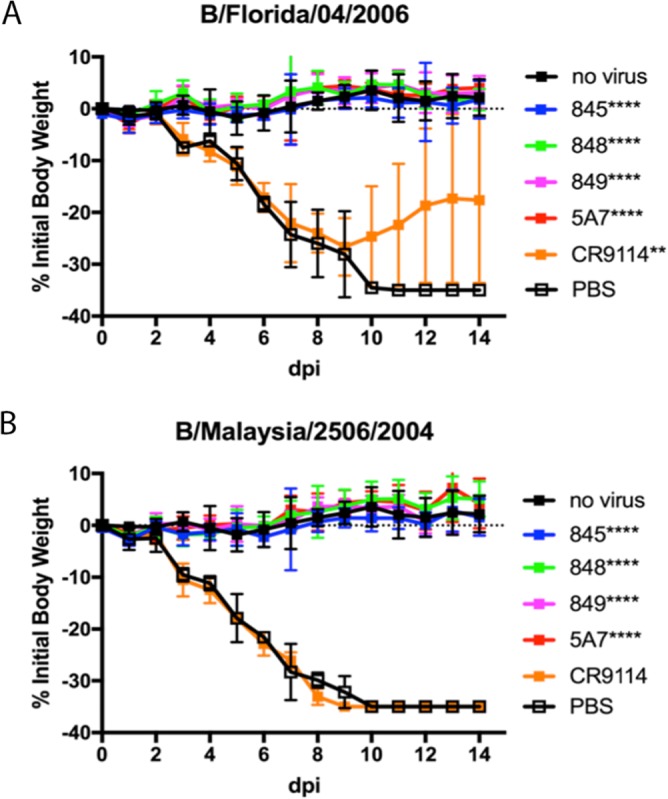
MAbs exhibit *in vivo* activity against both Yamagata and Victoria lineage strains. MAbs TRL845, TRL848, TRL849, 5A7, and CR9114 were tested for efficacy in a mouse influenza virus challenge model against B/Florida/04/2006 (Yamagata lineage) (A) and B/Malaysia/2506/2004 (Victoria lineage) (B). At 24 h after intranasal infection using a viral inoculum of 3 times the LD_50_ per mouse, each MAb was administered at 1 mg/kg MAb via the intranasal route (*n* = 5). Percent reductions in body weights are shown, with error bars being set at 1 standard deviation. dpi, days postinfection. ****, *P* < 0.0001; **, *P* < 0.005.

The MAbs were tested for their ability to protect mice from weight loss and lethality in a BALB/c mouse model of IBV (35). MAbs were administered at 1 mg/kg of body weight by the intranasal route 24 h after infection with IBV strain B/Florida/04/2006 (Yamagata lineage) or B/Malaysia/2506/2004 (Victoria lineage) (Fig. 4). Area-under-the-curve (AUC) analyses of the weight loss profiles indicated that treatments with all TRL MAbs and MAb 5A7 (28) were significantly differentiated relative to phosphate-buffered saline (PBS) treatments (*P* < 0.0001) and that all treatments resulted in 100% survival (log-rank test for survival, *P* < 0.005). In contrast, CR9114 (14) treatments against both infections were less effective, with substantial weight losses being observed and with only 3 and 0 mice surviving the B/Florida/04/2006 and B/Malaysia/2506/2004 infections, respectively.

**FIG 5:**
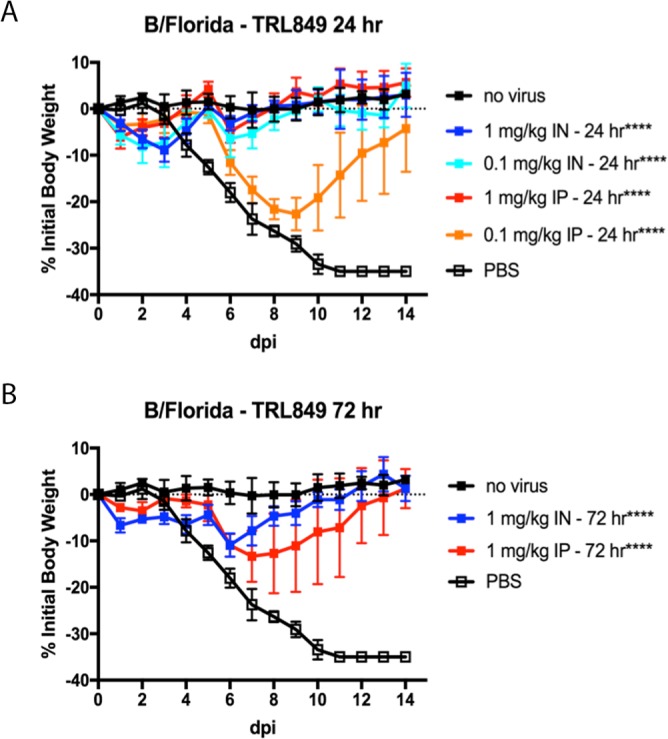
MAb TRL849 exhibits *in vivo* activity when administered at 24 or 72 h postinfection. A single treatment with TRL849, either intranasal (IN) or intraperitoneal (IP), was administered at the indicated doses at either 24 h (A) or 72 h (B) postinfection with Yamagata lineage strain B/Florida/04/2006 (*n* = 5). Percent reductions in body weights are shown, with error bars being set at 1 standard deviation. dpi, days postinfection. ****, *P* < 0.0001.

A dose-ranging experiment using TRL849 to treat B/Florida/04/2006 infection was performed (Fig. 5). AUC analyses of the weight loss profiles indicated that all MAb treatments were significantly differentiated relative to PBS treatments (*P* < 0.0001). All MAb-treated mice survived (log-rank test for survival, *P* < 0.005). Mice treated intraperitoneally with TRL849 at 1.0 mg/kg at 24 h postinfection were fully protected and showed a <10% transient weight loss, while mice receiving 0.1 mg/kg experienced >20% weight loss before recovering. Intranasal administration improved the potency, with 0.1 mg/kg at 24 h providing full protection from lethality and <10% weight loss. The increased potency of intranasal administration of neutralizing MAbs can be attributed to the increased levels of virus neutralization locally at the apical side of the site of infection, resulting in a lower viral burden (36, 37). When the 1.0-mg/kg treatment was administered at 72 h postinfection by either route of administration, transient weight loss of ∼10% and full protection from lethality were observed.

## DISCUSSION

Compared to mice immunized for a hybridoma antibody discovery effort, the human donors of the B cells that gave rise to the antibodies in this study were likely exposed to multiple IBV strains over time. While this situation might be expected to give rise to broadly cross-reactive anti-IBV antibodies, this outcome might also be considered unexpected, considering demonstrations that prior exposure can limit the responses to new variants, i.e., original antigenic sin (38). These two concepts might be reconciled considering a hierarchy of responses over time which still allow for the generation of broadly reactive antibodies (39).

Because the globular head region of HA is highly immunogenic, the frequency of high-affinity HA-specific antibodies reactive to the conserved stalk region epitopes was markedly lower, requiring a high-throughput technology to achieve a comprehensive screen of the human repertoire. As shown in Table 1, the set of 23 MAbs isolated in this study using the CellSpot technology is highly diverse, being derived from many different human germ line antibody sequences. The subset of 20 MAbs that we analyzed in detail is also notable in that several distinct classes are represented, including 11 nonneutralizers that bound to the stalk region and 9 neutralizers, with 4 HI-positive MAbs presumably binding to the head region and 5 binding to the stalk region. The fact that the set of 20 MAbs does not include any nonneutralizers binding to the head region likely reflects the screening process requirement that the MAbs must bind to HAs from both B/Florida/04/2006 (Yamagata lineage) and B/Malaysia/2506/2004 (Victoria lineage), representing divergent IBV strains. This screening constraint may have eliminated MAbs binding to nonconserved HA head sites that do not perform an essential viral function (such as binding to sialic acid on the host receptor), resulting in the absence of head-binding MAbs that are unable to neutralize the virus.

The multiplexed screening strategy employed in this study was designed to enrich for MAbs that bound to conserved sites on the HAs from diverse IBV strains. Mapping of the epitope data from the top four unique neutralizing MAbs to an alignment of the HA sequences derived from 2,543 unique IBV human isolates confirmed that these MAbs bind to very highly conserved IBV HA stalk residues (Fig. 3). Consistent with its essential role in the event of viral fusion with the host cell, the HA stalk region does not tolerate many mutations, making the discovery of MAbs that bind to the HA stalk an attractive and successful strategy for isolating broadly neutralizing anti-influenza virus antibodies (29, 40). Even though mutant IAV with certain amino acid substitutions in the HA stalk region that allow escape from neutralization by stalk-binding MAbs have been isolated, these IAV escape variants exhibited decreased viral fitness (41). Vaccine strategies that focus the immune response to the HA stalk region by employing a series of chimeric HA antigens in which the stalk region is held constant while the head region of the antigen is varied in successive booster doses have been successful in animals studies for IAV (42) and IBV (43). Stable headless HA vaccine molecules consisting of only the stalk region can also elicit broadly neutralizing antibodies to IAV (44). However, the ability of MAbs to bind to the HA stalk by itself may not ensure a neutralization phenotype, as evidenced by the 11 nonneutralizing HI-negative binders isolated in this study, some of which presumably bind to conserved epitopes on the HA stalk, given the initial selection criterion of binding HA from both lineages. Targeting other regions besides the stalk can be productive, as recent work has shown that broadly cross-reactive anti-IBV MAbs to the conserved regions of the sialic acid-binding region of the HA head (45) as well as the neuraminidase (46) can be generated in mice.

The human-derived HA stalk-binding antibodies isolated in this study bind HAs from a broad range of IBV strains (Table 3) and also show potent neutralization activities against IBV strains isolated from humans over many years (Table 4). They also show excellent activities against both IBV lineages in *in vivo* murine models of influenza virus infection (Fig. 4 and 5), with the activities comparing favorably to the activities observed for broadly neutralizing stalk-binding MAbs against IBV that have been previously described, notably, 5A7 (28) and CR9114 (14). Four of the five MAbs in this study exploit a varied set of epitopes in the HA stalk, as evidenced by their unique peptide signatures in the CLIPS analysis (Fig. 2 and 3) which correspond to highly conserved IBV HA residues (Fig. 3). The results of the CLIPS analysis for 5A7 are in excellent agreement with the previously published epitope map on the IBV stalk region for 5A7 determined by HA truncations and site-directed mutagenesis (28), which underscores the validity of the epitope mapping results obtained with the CLIPS methodology (33). While in some cases some residues in the CLIPS peptide signatures are shared with those of the previously described MAbs 5A7 and CR9114, the germ line sequences of all the MAbs included in this analysis (Fig. 2 and Table 1) are highly divergent among themselves, with each MAb representing a unique solution to binding to the IBV HA stalk and achieving viral neutralization. For instance, while TRL849 and CR9114 share some (but not all) of the same epitope space, as determined by CLIPS analysis (Fig. 2 and 3), their germ line origins are completely different and their *in vivo* activity profiles are highly differentiated, with TRL849 being much more efficacious against viral challenges by both Yamagata and Victoria lineage IBV strains (Fig. 4).

Clearly, the human antibody repertoire is a rich source capable of giving rise to diverse HA-binding antibodies that are able to neutralize IBV. The MAbs discovered in this study exhibit a combination of broad reactivity across IBV lineages, high potency, marked efficacy at 72 h postinfection, high thermal stability that generally correlates with ease of manufacturing, and the safety advantages of being derived directly from humans. These attributes make the novel MAbs suitable for use in investigative studies to assess their effectiveness in combating the morbidity and mortality due to IBV infections.

## MATERIALS AND METHODS

### Single B cell MAb discovery technology (CellSpot).

Leucopacks were obtained from a total of 8 anonymized donors under informed consent approved by Stanford's Institutional Review Board (Stanford Blood Center, Stanford, CA). Peripheral blood mononuclear cells (PBMCs) were prepared by standard methods, and individual memory B cells (CD19^+^/CD27^+^) were assayed following stimulation to proliferate and differentiate using a cocktail of mitogens and cytokines as previously described (23–26). The cells were distributed in 96-well microplates at ∼200 memory B cells/well. From 10 ml of blood, ∼100,000 memory B cells began a course of dividing and secreting IgG lasting ∼10 days. After 5 days, an aliquot of each well was transferred to a replicate microplate whose surface had been coated with an anti-Ig capture antibody; after gently spinning down the cells to the surface, a secreted IgG footprint of each cell was collected for 5 h and the cells were removed. These purified products of single cells were probed using homologous HA proteins from different strains conjugated, using sodium cyanoborohydride, to distinguishable fluorescent beads (6 types having different ratios of embedded red and green fluorophores). A lack of binding to beads coated with bovine serum albumin was used as a specificity counterscreen. This assay format provides replicates in the primary screen (sibling cells derived from the original positive cell in the master plate), which ensures a low false-positive rate. After identifying wells with B cells secreting a MAb meeting the selection criteria (typically, 1 or 2 wells per plate), the corresponding wells of the master plate were distributed at limiting dilution across new microplates. The secreted footprint was again collected, but without removal of the cells before assay. Although the signal in this secondary assay was weaker than that in the primary assay, the assay is nondestructive, and thus, the mRNAs encoding heavy and light chains could be amplified by a single-cell RT-PCR. After cloning into the pTT5 vector (47), recombinant antibodies were produced in HEK293 Freestyle cells (Thermo Fisher Scientific, Waltham, MA) by transient transfection and purified using protein A (MAb Select Sure; GE Healthcare, Pittsburgh, PA). The control MAbs 5A7 and CR9114 were similarly produced by cloning synthetic DNAs encoding the sequences of the heavy and light chain variable regions (as described previously [14, 28]) into recombinant expression vectors.

### Enzyme-linked immunosorbent assay (ELISA).

For analysis of recombinant antibody binding, 96-well microplates were coated with 2 μg/ml HA overnight in PBS at 4°C. HA proteins were purchased from Sino Biological for B/Florida/04/2006 (catalog number 11053-V08H), from Protein Sciences for B/Malaysia/2506/2004 (catalog number /2506/04), and from Immune Tech for B/Brisbane/60/2008 (catalog number IT-003-B2TMp), B/Victoria/2/1987 (catalog number IT-003-B6p), B/Massachusetts/02/2012 (catalog number IT-003-B19pTMp), and B/Wisconsin/1/2010 (catalog number IT-003-B7TMp). On the next day, the plates were blocked with 3% bovine serum albumin (BSA) in PBS and then incubated for 1 h with serial dilutions of the MAb starting at 5 μg/ml (30 nM) in 0.5% BSA in PBS-Tween 20 (PBS-T). After washing with PBS-T, horseradish peroxidase-conjugated anti-human immunoglobulin kappa chain was added for 45 min. The plates were then washed with PBS-T and developed with SureBlue tetramethylbenzidine substrate (KPL Inc., Gaithersburg, MD).

### Virus neutralization assay.

The IBV-neutralizing activity of the MAbs was titrated to determine 50% inhibitory concentration (IC_50_) values in a 72-h plaque formation assay on MDCK cells (CCL-34; ATCC) as described previously (34). On the day before the assay, one 12-well plate per test antibody was seeded with 2 × 10^5^ MDCK cells/well in minimal essential medium (MEM)–5% fetal bovine serum–1% l-glutamine. Virus stocks whose titers were predetermined were rapidly thawed and diluted in MEM–1% l-glutamine and 1 μg/ml tosylsulfonyl phenylalanyl chloromethyl ketone (TPCK) trypsin (Worthington, Lakewood, NJ), vortexed, and kept on ice. Antibodies were added to the virus at concentrations ranging from 0 to 20 μg/ml, vortexed, and incubated at 37°C for 1 h. Cell monolayers were washed 3 times with PBS, and 100 μl MEM–1% l-glutamine and 1 μg/ml TPCK trypsin was added to each well before 200 μl of the preincubated virus-antibody preparation was added. After 2 h at 37°C, the supernatant was aspirated, the monolayer was washed once with PBS, and a 1.2% Avicel RC-581 (FMC Bio-polymer Inc., Philadelphia, PA) overlay containing 1 μg/ml TPCK trypsin in MEM, 20 mM HEPES, 2 mM l-glutamine, 0.075% NaHCO_3_, 100 units/ml penicillin G, 100 μg/ml streptomycin, and 0.25 μg/ml amphotericin B was applied for 72 h at 37°C in 5% CO_2_. The plaques were visualized by fixing and staining with crystal violet. First, the Avicel overlay was aspirated and the monolayers were gently washed with PBS. The monolayers were then fixed by applying 1 ml of ice-cold 80% methanol–20% acetone for 10 min. Stock solutions of 0.13% (wt/vol) crystal violet were prepared in deionized water containing 5% methanol and 11% formaldehyde. Working stocks of crystal violet were made by diluting the stock solution 1:2 in PBS. After monolayer fixation, enough crystal violet working solution was added to a well to just cover the cells, and the cells were allowed to stain for 10 to 20 min. The wells were rinsed in distilled deionized water, and the average number of plaques from duplicate wells was counted and expressed as a percentage of the plaques in the uninhibited (100% infected) control wells. IC_50_s were calculated using the log (inhibitor) versus response nonlinear regression analysis method within the Prism (version 7) software package (GraphPad Software, Inc., La Jolla, CA).

### Hemagglutination inhibition assay.

The hemagglutination inhibition (HI) assay was performed as described previously (30) using strains B/Victoria/2/1987 and B/Yamagata/16/1988.

### Thermal stability assay.

The melting temperatures (*T_m_*) of the MAbs were determined by differential scanning fluorimetry (31). Reactions were run in a StepOne Plus instrument (Life Technologies, Carlsbad, CA). Antibodies were diluted to 2 μg/ml into PBS containing 1× SYPRO Orange (Life Technologies, Carlsbad, CA) in a final volume of 20 μl. The reaction tubes were heated for 2 min at 25°C, followed by a 1% ramp rate (∼0.8°C/min) to 99°C and 2 min at 99°C. The fluorescent intensity of the emission signal was monitored over the course of the incubation using the filters for the carboxy-X-rhodamine dye (Thermo Fisher Scientific, Waltham, MA). Melting temperatures were determined by curve fitting the first derivative of the fluorescence data in Microsoft Excel software and confirmed, using Protein Thermal Shift software (version 1.0; Life Technologies, Carlsbad, CA), using the derivative *T_m_* method. All plots were made using Prism software (GraphPad Software, Inc., La Jolla, CA). *T_m_* values were determined from the average of 3 or more experiments, each of which was run in duplicate.

### Affinity measurement.

A stock solution of MAb at 600 nM was diluted to 5.0 and 0.5 nM in PBS (pH 7.4), and affinities were measured with a ForteBio Octet biosensor (model QK; Pall Corporation, Menlo Park, CA). Streptavidin-coated sensors were dipped into biotinylated HA antigen (1 mg/ml in PBS, pH 7.4) after a wash step with the MAbs at a concentration of 250 nM. The resulting on and off rates measured yielded the *K_D_* by standard methods (48).

### Epitope mapping.

Using the chemical linkage of peptides onto scaffolds (CLIPS) technology (Pepscan B.V., Lelystad, Netherlands), a set of >6,500 peptides covering linear, discontinuous, and conformational epitopes (including single loops, double loops, triple loops, sheet-like folds, helix-like folds, and combinations thereof) was designed and synthesized as previously reported (33) using HA2 from influenza B/Lee/40/1940. ELISA binding assays were used to identify MAb-binding sites, which were visualized by projection onto the structure of the strain B/Lee/40/1940 influenza B virus HA (PDB accession number 4NRL).

### Bioinformatics.

All unique human-derived full-length IBV HA sequences deposited as of 31 December 2017 in the Influenza Research Database (IRD; http://www.fludb.org) were obtained (2,543 unique HA sequences). Using the IDB set of analysis tools (http://www.viprbrc.org), the HA sequences were aligned and an analysis was performed to determine a positional entropy score (related to the Shannon entropy) at each aligned sequence position. The entropy scores at each HA residue position range from zero (100% conservation) to 232 (a random distribution of all 20 amino acid residue types). These values were used to assess the conservation at each of the IBV HA residue positions making up the epitopes recognized by the anti-IBV MAbs.

### Mouse model.

Female BALB/c mice were ordered from The Jackson Laboratory and were 6 to 8 weeks old at the time of the experiments. Mice (*n* = 5 per group) were anesthetized with a ketamine-xylazine mixture and subsequently infected intranasally using 50 μl of PBS containing 3 times the 50% lethal dose (LD_50_) of either B/Florida/04/2006 (4,500 PFU) or B/Malaysia/2506/2004 (13,000 PFU). The anesthetized mice were treated at 24 or 72 h postinfection with 0.1 or 1.0 mg/kg body weight of MAb in PBS delivered as a 0.1-ml bolus by intraperitoneal injection or as 0.05 ml by intranasal instillation. The mice were monitored for death, morbidity, and weight loss, and the date of death was recorded for 14 days.

### Statistical analysis.

All statistical analyses and plot generations were made using Prism (version 7) software (GraphPad Software, Inc., La Jolla, CA). Area-under-the-curve (AUC) analysis was used to assess the statistical relationship between the percent weight loss profiles of the different treatments. Areas under the weight loss curves were measured using the AUC functionality of Prism (version 7) software. The statistical relationship associated with any two weight loss curves was assessed by calculating the mean AUC, the standard error, and the number of degrees of freedom for each pair and performing the unpaired *t* test. Survival data were analyzed by the log-rank test.
